# Supplementary material for: Cancer prevention recommendations: awareness in a Mexican public hospital
Source: PeerJ. 2024 Jul 11;12:e17593. doi: 10.7717/peerj.17593 (PMC11246616; doi:10.7717/peerj.17593)
Supplement: Supplemental Information 1 [file peerj-12-17593-s001.docx]

Supplementary file A

Questionnaire used.

1. For women: Does maintaining a waist circumference less than 80 centimeters prevent cancer? a) **Yes**, b) No, or c) I don´t know
2. For men: Does maintaining a waist circumference of less than 94 centimeters prevent cancer? a) **Yes**, b) No, or c) I don´t know
3. Does remaining at a healthy weight (i.e., not being overweight or obese) in adulthood prevent cancer? a) **Yes**, b) No, or c) I don´t know
4. Does not smoking cigars, pipes, or cigarettes prevent cancer? a) **Yes**, b) No, or c) I don´t know
5. Does having sedentary behavior, such as watching a lot of television, prevent cancer? a) Yes, b) **No**, or c) I don´t know
6. Does performing vigorous exercise or physical activity that makes you breathe harder than normal, such as aerobics, fast bicycling, playing soccer, running, swimming, or heavy agricultural work, such as harvesting, for 30 minutes each day prevent cancer? a) **Yes**, b) No, or c) I don´t know
7. Does consuming foods with a lot of calories, such as french fries, cookies, cupcakes, fried foods, or hamburgers, prevent cancer? a) Yes, b) **No**, or c) I don´t know
8. Does drinking beverages with high sugar content, such as soft drinks, prevent cancer? a) Yes, b) **No**, or c) I don´t know
9. Does consuming 400 grams of vegetables or fruits daily prevent cancer? To guide you in your answer, the following are the average weights of some vegetables and fruits: tomato, 120 grams; apple, 138 grams; banana, 80 grams; carrot, 64 grams; mango, 200 grams; and mandarin, 180 grams. a) **Yes**, b) No, or c) I don´t know
10. Does consuming less than 500 grams (half a kilogram) of beef, pork, or lamb a week, equivalent to approximately three times per week at most, prevent cancer? a) **Yes**, b) No, or c) I don´t know
11. Does consuming little or no ham, sausage, pork cheese, bacon, chorizo, or salami prevent cancer? a) **Yes**, b) No, or c) I don´t know
12. For women: Does consuming one glass of an alcoholic drink per day or less prevent cancer? To guide you in your answer, a glass of beer is equivalent to ≤ 375 ml; wine, ≤ 150 ml; liquor, ≤ 75 ml; and spirits, ≤ 45 ml. a) **Yes**, b) No, or c) I don´t know
13. For men: Does consuming two glasses of an alcoholic drink per day or less prevent cancer? To guide you in your answer, two glasses of beer is equivalent to ≤ 750 ml; wine, ≤ 300 ml; liquor, ≤ 150 ml; and spirits, ≤ 90 ml. a) **Yes**, b) No, or c) I don´t know
14. Does consuming foods with high salt content or preserved with salt, such as cod, shrimp, charal, salted meat, machaca, and serrano ham, prevent cancer? a) Yes, b) **No**, or c) I don´t know
15. Does consuming nutritional supplements, such as pills, powders, syrups, and drinks (e.g., Ensure or Glucerna), prevent cancer? a) Yes, b) **No**, or c) I don´t know
16. Does feeding babies only breast milk from birth to six months prevent cancer? a) **Yes**, b) No, or c) I don´t know
17. Do you believe that cancer is a disease that can be prevented or avoided? a) **Yes** or b) No
18. What do you think is the main cause of cancer? a) Heredity or b) **Lifestyle**
19. In the last week, how often did you smoke cigarettes, a pipe, or a cigar? a) Always, b) Almost always, c) Sometimes, d) Almost never, or e) Never
20. For women: In the last week, how often did you have more than one glass of an alcoholic beverage per day? To guide you in your answer, a glass of beer is equivalent to ≤ 375 ml; wine, ≤ 150 ml; liquor, ≤ 75 ml; and spirits, ≤ 45 ml. a) Always, b) Almost always, c) Sometimes, d) Almost never, or e) Never
21. For men: In the past week, how often did you have more than two glasses of alcoholic beverages per day? To guide you in your answer, two glasses of beer is equivalent to ≤ 750 ml; wine, ≤ 300 ml; liquor, ≤ 150 ml; and spirits, ≤ 90 ml. a) Always, b) Almost always, c) Sometimes, d) Almost never, or e) Never

Note: For questions 1–18, the correct answers are indicated in bold.
