# Supplementary material for: Cancer prevention recommendations: awareness in a Mexican public hospital
Source: PeerJ. 2024 Jul 11;12:e17593. doi: 10.7717/peerj.17593 (PMC11246616; doi:10.7717/peerj.17593)
Supplement: Supplemental Information 2 [file peerj-12-17593-s002.docx]

**Cuestionario usado**

1. Para mujeres: ¿Mantener una circunferencia de la cintura inferior a 80 centímetros previene el cáncer? a) Sí, b) No, c) No sé

2. Para hombres: ¿Mantener una circunferencia de la cintura inferior a 94 centímetros previene el cáncer? a) Sí, b) No, c) No sé

3. ¿Permanecer en un peso saludable (es decir, no tener sobrepeso, ni ser obeso) en la edad adulta previene el cáncer? a) Sí, b) No, c) No sé

4. ¿No fumar cigarro, pipa o puro previene el cáncer? a) Sí, b) No, c) No sé

5. ¿Tener costumbres sedentarias, como mirar mucha televisión, previene el cáncer? a) Sí, b) No, c) No sé

6. ¿Realizar ejercicio o actividad física vigorosa que hacen que la persona respire con mayor dificultad de lo normal, como los aeróbicos, andar en bicicleta rápidamente, jugar futbol, correr, nadar o realizar trabajo agrícola pesado, como cosechar, por 30 minutos diariamente previene el cáncer? a) Sí, b) No, c) No sé

7. ¿Consumir alimentos con mucha energía como las papas fritas, las galletas, los pastelitos, las frituras o las hamburguesas previene el cáncer? a) Sí, b) No, c) No sé

8. ¿Tomar bebidas con alto contenido de azúcar como los refrescos previene el cáncer? a) Sí, b) No, c) No sé

9. ¿Consumir 400 gramos de verduras o frutas al día previene el cáncer? Para orientarle en tu respuesta los pesos promedio de algunas verduras y frutas son tomate, 120 gramos; manzana, 138 gramos; plátano, 80 gramos; zanahoria, 64 gramos; mango, 200 gramos; y mandarina, 180 gramos. a) Sí, b) No, c) No sé

10. ¿Consumir menos de 500 gramos (medio kilo) de carne de res, puerco o borrego a la semana, esto es aproximadamente consumir este tipo de alimentos como máximo 3 veces por semana, previene el cáncer? a) Sí, b) No, c) No sé

11. ¿Consumir poco o nada de jamón, salchicha, longaniza, queso de puerco, tocino, chorizo o salami previene el cáncer? a) Sí, b) No, c) No sé

12. Para mujeres: ¿Consumir un vaso de bebida alcohólica al día o menos previene el cáncer? Para orientarle en su respuesta, un vaso de cerveza equivale a ≤ 375 ml; vino, ≤ 150 ml; licor, ≤ 75 ml; y licores, ≤ 45 ml. a) Sí, b) No, c) No sé

13. Para hombres: ¿Consumir dos vasos de bebida alcohólica al día o menos previene el cáncer? Para orientarle en su respuesta, dos vasos de cerveza equivalen a ≤ 750 ml; vino, ≤ 300 ml; licor, ≤ 150 ml; y licores, ≤ 90 ml. a) Sí, b) No, c) No sé

14. ¿Consumir alimentos con alto contenido de sal o preservados en sal como bacalao, camarón, charal, carne salada, machaca y jamón serrano, previene el cáncer? a) Sí, b) No, c) No sé

15. ¿Consumir suplementos nutricionales o alimenticios, como pastillas, polvos, jarabes, bebidas como ensure o glucerna, previene el cáncer? a) Sí, b) No, c) No sé

16. ¿Alimentar a los bebés únicamente con leche materna desde el nacimiento hasta los seis meses previene el cáncer? a) Sí, b) No, c) No sé

17. ¿Cree que el cáncer es una enfermedad que se puede prevenir o evitar? a) Sí o b) No

18. ¿Cuál cree que es la principal causa del cáncer? a) Herencia b) Estilo de vida

19. En la última semana, ¿con qué frecuencia fumó cigarrillos, pipa o puro? a) Siempre, b) Casi siempre, c) A veces, d) Casi nunca, o e) Nunca

20. Para mujeres: En la última semana, ¿con qué frecuencia tomó más de un vaso de bebida alcohólica al día? Para orientarle en su respuesta, un vaso de cerveza equivale a ≤ 375 ml; vino, ≤ 150 ml; licor, ≤ 75 ml; y licores, ≤ 45 ml. a) Siempre, b) Casi siempre, c) A veces, d) Casi nunca, o e) Nunca

21. Para hombres: En la última semana, ¿con qué frecuencia consumió más de dos vasos de bebidas alcohólicas al día? Para orientarle en su respuesta, dos vasos de cerveza equivalen a ≤ 750 ml; vino, ≤ 300 ml; licor, ≤ 150 ml; y licores, ≤ 90 ml. a) Siempre, b) Casi siempre, c) A veces, d) Casi nunca, o e) Nunca
