# Supplementary material for: Cancer prevention recommendations: awareness in a Mexican public hospital
Source: PeerJ. 2024 Jul 11;12:e17593. doi: 10.7717/peerj.17593 (PMC11246616; doi:10.7717/peerj.17593)
Supplement: Supplemental Information 4 [file peerj-12-17593-s004.docx]

**Codebook to convert numbers to their respective factors (categorical data)**

**Homemarker**

0: Yes

1: No

**Skills**

0: No

1: Yes

**Poverty**

0: Yes

1: No

**Questions 1-16**

0: Incorrect

1: Correct

**Groups**

1: Group A

0: Group B

**Question 17**

0: Smoke

1: No smoke

**Question 18**

0: Drink

1: No drink
