# Supplementary material for: Cancer prevention recommendations: awareness in a Mexican public hospital
Source: PeerJ. 2024 Jul 11;12:e17593. doi: 10.7717/peerj.17593 (PMC11246616; doi:10.7717/peerj.17593)
Supplement: Supplemental Information 6 [file peerj-12-17593-s006.docx]

Supplementary file B

Coefficients, confidence intervals, and p-values of the multiple regression analysis (n = 289).

| **Variable** | **Coefficient** | **Lower limit *** | **Upper limit **** | **P-value** |
| --- | --- | --- | --- | --- |
| Women | 2.140 | 2.037 | 2.244 | < 0.001 |
| Men | 2.184 | 2.067 | 2.301 | < 0.001 |
| Homemaker | 0.126 | 0.007 | 0.244 | 0.039 |
| Belong to Group A | 0.069 | 0.028 | 0.111 | 0.001 |
| Age | 0.004 | 0.002 | 0.006 | < 0.001 |
| Education ≥ 12 years | 0.063 | 0.013 | 0.112 | 0.014 |
| Homemaker: age | -0.002 | -0.005 | 0.000 | 0.080 |

Group A: participants who answered that cancer is preventable through lifestyle. * 95% confidence interval
